# Role of intestinal flora in the development of nonalcoholic fatty liver disease in children

**DOI:** 10.1128/spectrum.01006-23

**Published:** 2024-01-08

**Authors:** Jing Zhang, Mengxuan Shi, Chunna Zhao, Guangcai Liang, Chuan Li, Xiaomeng Ge, Caixia Pei, Yawei Kong, Dongdan Li, Wenli Yang, Bingyan Cao, Libing Fu, Yinkun Yan, Jie Wu, Jin Zhou, Yongli Fang, Xi Meng, Yong Li, Liming Wang

**Affiliations:** 1 National Center for Children’s Health, Beijing Children’s Hospital, Capital Medical University, Beijing, China; 2 CAS Key Laboratory of Microbial Physiological and Metabolic Engineering, State Key Laboratory of Microbial Resources, Institute of Microbiology, Chinese Academy of Sciences, Beijing, China; 3 Department of Scientific Research, Microvita Medical Technology Co., Beijing, China; 4 Microbial Resources and Big Data Center, Institute of Microbiology, Chinese Academy of Sciences, Beijing, China; 5 Department of Thoracic Surgery, National Cancer Center/National Clinical Research Center for Cancer/Cancer Hospital, Chinese Academy of Medical Sciences and Peking Union Medical College, Beijing, China; Huazhong University of Science and Technology, Wuhan, China; National Taiwan University Hospital, Taipei, Taiwan

**Keywords:** BMI, children, gut microbiota, metabolism, NAFLD, NASH

## Abstract

In China, 45% of adolescents with obesity develop fatty liver disease, a condition that increases the long-term risk of developing cirrhosis and liver cancer. Although the factors triggering nonalcoholic fatty liver disease (NAFLD) vary in children, the composition of intestinal microflora has been found to play an increasingly important role. However, evidence is limited on the prevalence of nonalcoholic fatty liver (NAFL) and nonalcoholic steatohepatitis (NASH) in Chinese children. Therefore, this study aimed to evaluate the fecal microbiome of Chinese children with NAFLD and further analyze the potential of flora in regulating NAFLD-related symptoms and metabolic functions. Specifically, the study applied a 16S rRNA and metagenomic sequencing to the fecal samples of pediatric patients with NAFLD, NASH, and NAFL, as well as healthy controls, to explore the correlation among NAFLD-related indexes, metabolic pathways, and gut flora. The findings showed that some fecal microbiota had a negative correlation with body mass index, and various NAFLD-related bacteria, including *Lachnoclostridium*, *Escherichia-Shigella*, and *Faecalibacterium prausnitzii*, were detected. Consequently, the study concluded that the variation in gut microbiota might be more important in improving NAFLD/NASH compared with single species, providing a microbiota diagnostic profile of NAFLD/NASH.

**IMPORTANCE:**

This study aims to characterize the gut microbiota in Chinese children with nonalcoholic fatty liver disease (NAFLD) through 16S rRNA and metagenomic sequencing. The results highlight the association between fecal microbiota and NAFLD in Chinese children, demonstrating distinct characteristics compared to adults and children from other countries. Based on the sequencing data from our cohort’s fecal samples, we propose a microbiota model with a high area under the curve for distinguishing between NAFLD and healthy individuals. Furthermore, our follow-up study reveals that changes in the relative abundance of microbial biomarkers in this model are consistent with variations in patients’ body mass index. These findings suggest the potential utility of the microbiota model and microbial biomarkers for diagnosing and treating NAFLD in children.

## INTRODUCTION

Over the past two decades, the incidence of nonalcoholic fatty liver disease (NAFLD) has more than doubled among children and adolescents globally, emerging as the most prevalent cause of chronic liver disease in various nations (1). In China, it is estimated that 45% of adolescents with obesity have NAFLD (2). According to Chinese and US clinical guidelines, children with NAFLD are more likely to develop serious diseases in adulthood, such as hypertension and liver cancer (3). NAFLD in children comprises two distinct pathological states—nonalcoholic fatty liver (NAFL) and nonalcoholic steatohepatitis (NASH)—based on the classification of liver biopsy findings (4). Patients with NASH face reduced survival due to cardiovascular and liver-related causes (5).

Although the factors contributing to the severity of the disease remain poorly understood, the composition of gut microbiota has emerged as a pivotal element in NAFLD pathophysiology in children. The gut microbiome comprises trillions of microbial organisms that exist symbiotically with the host, providing critical immune development, mucosal protection, and nutrient functions (1). Previous research suggests that the gut microbiome may influence the processes involved in the onset and progression of NAFLD, such as choline metabolism, obesity, endotoxemia, and liver inflammation or fibrosis (2–4). As shown in Table 1, the characterization of NAFLD/NASH gut microbiotas demonstrated varying age- and country-specific patterns.

**TABLE 1:** Studies of microbiome differentiation in patients with NAFLD

Studies	Groups	Methods	Microbiome
Lee et al., 2020 (1)	171 patients with NAFLD and 31 non-NAFLD controls (adult Korean)	Fecal microbiome: 16S rRNA	NAFLD vs healthy control (HC): *Faecalibacterium*↓ Ruminococcaceae↓ Lachnospiraceae↓ *Coprococcus*↓ Rikenellaceae↑ Enterobacteriaceae↑ *Citrobacter*↑
Adams et al., 2020 (2)	67 patients with NAFLD and 55 non-NAFLD controls (adult Australian)	Fecal microbiome: 16S rRNA	NAFLD vs HC: Lachnospiraceae↑ Bacteroidaceae↓
Boursier et al., 2016 (3)	57 patients with NAFLD, including 35 with NASH (adult French)	Fecal microbiome: 16S rRNA	NASH vs NAFLD: *Bacteroides*↑ *Ruminococcus*↑ *Prevotella*↓
Marialena et al., 2013 (4)	11 patients with NAFLD, 22 with NASH, and 17 non-NAFLD controls (adult Canadian)	Fecal microbiome: quantitative real-time polymerase chain reaction	NASH vs NAFLD/HC: Bacteroidetes↓ *Clostridium coccoides*↑
Fei et al., 2020 (5)	32 patients with NAFLD and 36 non-NAFLD controls (pediatric Chinese)	Fecal microbiome: metagenome	NAFLD vs HC: Ruminococcaceae↓ *Faecalibacterium*↓
Monga et al., 2020 (6)	73 patients with NAFLD (pediatric American)	Fecal microbiome: 16S rRNA	HFF>5.5% vs HFF<5.5%: *Prevotella*↓ *Gemmiger*↓ *Oscillospira*↓
Schwimmer et al., 2019 (7)	87 patients with biopsy-proven NAFLD and 37 obese non-NAFLD patients (pediatric American)	Fecal microbiome: 16S rRNA	NASH vs NAFLD: *Faecalibacterium*↓ *Alistipes*↓ *Parabacteroides*↓
Federica et al., 2017 (8)	27 patients with NAFL, 26 with NASH, or 8 obese and 54 healthy controls (pediatric Italian)	Fecal microbiome: 16S rRNA	NASH vs HC: *Oscillospira*↓ *Dorea*↑ *Ruminococcus*↑ *Blautia*↑
Zhu et al., 2013 (9)	25 obese, 22 with NASH, and 16 healthy controls (pediatric American)	Fecal microbiome: 16S rRNA	NASH vs HC: Proteobacteria↑ *Escherichia* ↑ *Prevotella*↑ Lachnospiraceae↓ Ruminococcaceae↓

Despite studies of the relationship between NAFLD and gut bacteria in animal models and humans (7, 10), limited research has been conducted on this issue in children, particularly concerning NAFL and NASH. Hence, this study aimed to assess the fecal microbiome of Chinese children diagnosed with NAFLD, thereby enabling the exploration of the potential of gut flora in controlling disease-associated symptoms and metabolic functions.

## MATERIALS AND METHODS

### Participants

A total of 114 participants were recruited for the study between January 2019 and December 2021, comprising 79 patients with NAFLD and 35 healthy controls (HCs). The participants were aged 7–18 years, with information on various parameters such as sex, birth date, height, and weight collected from their professionally trained parents. Recruited in the NAFLD group were patients whose body mass index (BMI) was higher than the 95th percentile and defined through a clinical evaluation and liver ultrasonography (10). The BMI values were used to classify overweight and obesity among participants, with BMI calculated as the ratio of weight (kg) to height squared (m^2^). Participants with a recent history of antibiotic intake within 2 months or probiotics use within a month before enrollment were excluded. The liver function tests and lipid profile assessment were conducted on fasting blood samples, and the fecal samples were also collected (11). The diagnostic criteria for metabolic disorders of NAFLD were as follows (12): (i) central obesity or overweight; (ii) antidiabetic treatment or fasting glucose level ≥ 7.0 mmol/L; (iii) antihypertensive treatment or elevated collected blood pressure with ≥95th percentile in children of the same age and sex; (iv) treatment with lipid-lowering agents or triacylglycerols ≥ 1.47 mmol/L; and (v) treatment with lipid-lowering agents or high-density lipoprotein cholesterol < 1.1 mmol/L in men or <1.3 mmol/L in women. Fifteen children with NAFLD were reassessed for their BMI, and fecal samples for metagenomic sequencing were obtained following 3–6 months of nonmedical treatment such as diet control and increased exercise.

### Pathological assessment

The liver biopsies were collected from children whose parents or guardians provided consent for liver puncture to assess NAFLD activity score (NAS) and liver fibrosis stage. The liver tissues of 13 children were obtained through ultrasound-guided liver biopsy and fixed in formaldehyde. The specimens were embedded in paraffin, after which serial sectioning was performed, and specimens were stained with hematoxylin-eosin (HE) and reticular fiber and/or Masson trichrome staining. The slides were then examined under a light microscope. Two pathologists scored the pathological diagnosis results at the same time; in case of disagreement, a senior physician was invited. NAFL was assessed according to NAS diagnosis criteria of NAFLD on pathological liver tissue (or liver biopsy) (13). NAFL was defined as liver cell steatosis without any indication of steatohepatitis, with or without liver fibrosis; NASH was defined as the presence of hepatic steatosis with necroinflammation and hepatocellular injury with or without fibrosis. NAFLD with fibrosis can refer to either NAFL or NASH associated with fibrosis in port, periportal, perisinusoidal, or portal-portal bridging fibrosis (13). The diagnostic criteria for NAFLD and NASH, including the scoring rules for NAS, the stage of liver fibrosis, and the pathological diagnosis of NASH, refer to the Expert Consensus on the Diagnosis and Treatment of NAFLD in Children and the study by Kleiner et al. (13, 14).

**Fig 1:**
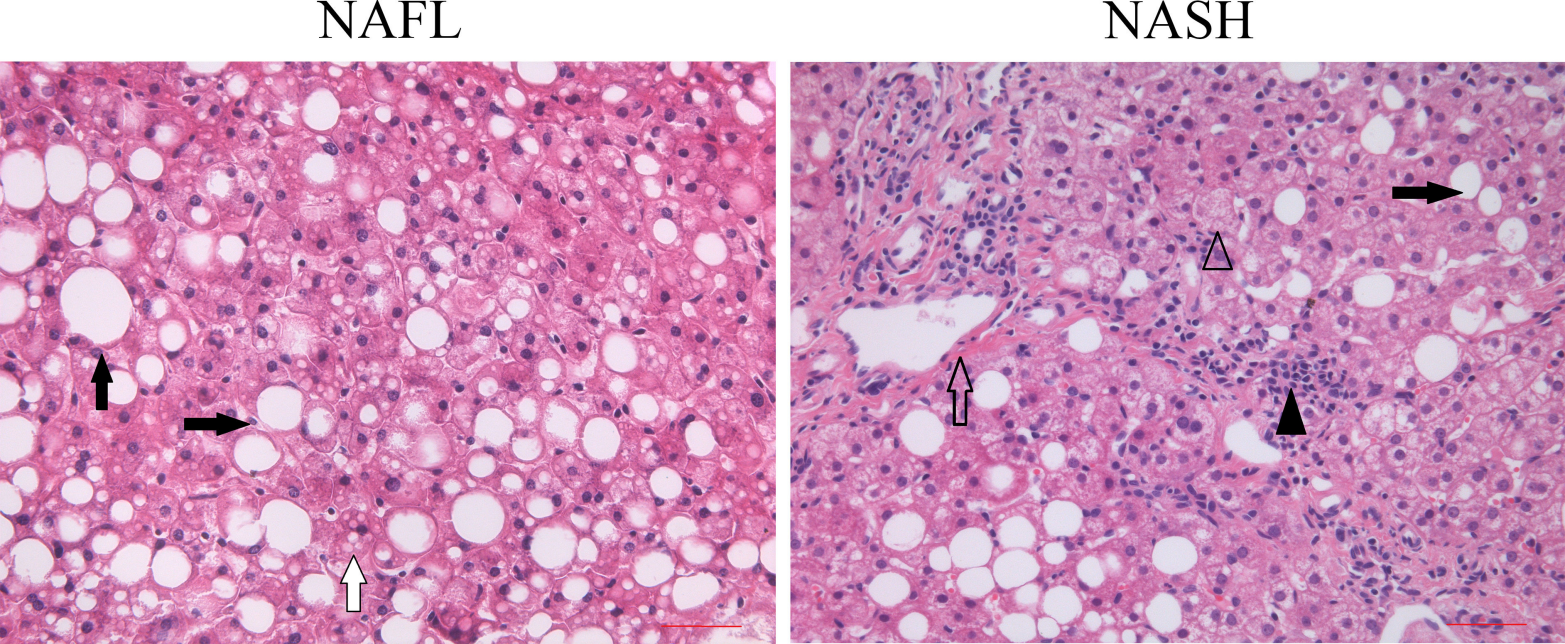
The histomorphological diagnosis of liver biopsy specimens in patients diagnosed with NAFL or NASH. The HE staining of the liver biopsy specimens of NAFL only shows steatosis. The HE staining of NASH shows steatosis, lobular inflammation, ballooning, and liver fibrosis. The black arrow, white arrow, black arrowhead, empty arrowhead, and empty arrow indicate macrovesicular steatosis, microvesicular steatosis, lobular inflammation, ballooning, and liver fibrosis, respectively. Scale bar, 250 µm. Images of all pathological sections were provided by the Pathological Department, Beijing Children’s Hospital.

Out of the 79 children diagnosed with NAFLD, our analysis included only 13 children for whom parental consent was obtained for liver puncture. Following the evaluation of the NAS, we identified eight cases of NASH and five cases of NAFL. As shown in Fig. 1, the NAS was created as an unweighted score for steatosis (0–3), lobular inflammation (0–3), and ballooning (0–2). A four-scale grading (from 0 to 3) was used for grading steatosis: normal liver (grade 0) contains fat in <5% of hepatocytes, while grade 1 steatosis refers to <33% steatotic hepatocytes. In grade 2 and 3 steatoses, fat is present in at least 33% or 66% of hepatocytes, respectively. The grading system of lobular inflammation was determined by the number of inflammatory foci in a given area, from grade 0 to 3 (0–3 points, none, <2, 2–4, and >4, respectively). The grading of ballooned cells was based mainly on number (0–2 points, none, few, and many, respectively). The total NAS score ranged from 0 to 8, where scores of 0–2 can exclude NASH, 3–4 suggested possible NASH, and scores of 5–8 were used to diagnose NASH. Additionally, the progression of fibrosis was described as follows (Fig. 1): zone 3 perivenular and perisinusoidal represented stage 1 (with a subdivision into 1a and 1b according to the amount of the deposit), and stage 1c was isolated periportal fibrosis; stage 2 included portal and central fibrosis without bridging fibrosis; stage 3 was bridging fibrosis; and stage 4 was cirrhosis.

### Biochemical data

After overnight fasting, 4 mL of peripheral blood was drawn from the cubital vein of each participant (15). The blood samples were centrifuged at 2,500 *g* for 10 min using an analyzer (KHB, Shanghai, China). The resulting supernatants were collected and stored for analysis. The serum levels of fasting blood glucose, uric acid, thyroid-stimulating hormone (TSH), alanine aminotransferase (ALT), and aspartate aminotransferase (AST) were measured using a Beckman Coulter AU5800 analyzer (CA, USA).

### Fecal sample collection and DNA extraction

The fecal specimens from participants were collected in sterilized 2 mL tubes containing a protective solution and frozen at −80°C until DNA extraction. Total DNA was extracted from the fecal samples (250 mg, wet weight) using a PSP Spin StooL DNA Kit/PSP Spin Stool DNA Plus Kit (Stratec Molecular GmbH, Berlin, Germany) following the manufacturer’s protocols (10).

### 16S rRNA sequencing and analysis

The stool samples were processed at MyGenostics Co. Ltd. (Beijing, China). The 16S rRNA amplicon sequencing of the V3–V4 gene region was used on an Illumina MiSeq platform to detect and quantify bacterial taxa in the fecal samples of HCs and children with NAFLD. The paired-end 16S sequences were merged and filtered for quality using the laser flash method. The Quantitative Insights into Microbial Ecology (QIIME, version 2.0) software suite was used to analyze the sequences, and the QIIME tutorial (http://qiime.org/) was followed with some modifications. The sequences were compared with the 97% reference data set of the SILVA database (SILVA project, version 138.1, http://www.arb-silva.de/). Those that did not match any reference sequences were clustered into *de novo* operational taxonomic units (OTUs) at 97% similarity using the UCLUST algorithm. This allowed us to accurately assign taxonomical classifications to our sequencing reads and perform downstream analyses with confidence. To obtain the population profiles of common and abundant taxa, we constructed a distance matrix based on the abundance of OTUs. Hierarchical clustering was performed using the unweighted pair group method with arithmetic mean (average linkage clustering) on this distance matrix. A Newick tree format was obtained using the QIIME package. Furthermore, QIIME was used to analyze the alpha diversity (observed species, Chao1, Shannon, and Simpson) and beta diversity [principal coordinate analysis (PCoA)]. Linear discriminant analysis effect size (LEfSe) was applied to compare significant differences in taxa between patients with NAFLD and HCs using a *P* value < 0.05.

In this study, we utilized the random forest supervised learning algorithm on 16S data sets to distinguish between HCs and NAFLD samples. We set the ntree parameter to 500 for the number of trees to construct. Two-thirds of all data were utilized as the training set, while the remaining third constituted the test set. To reduce the possibility of confounding variables due to correlated data, we normalized the filtered relative abundances. The model was trained using 114 stool samples, 35 of which were HC and 79 were NAFLD samples. The class error calculated using the model was 0.3714. The model accurately identified 22 HC samples. For the NAFLD samples, the model could predict all samples precisely. The estimated error rate of the model was 0.11404, while the baseline error for random guessing sat at 0.30702. The ratio of baseline error to observed error for our modeling was 2.69231. We used the R programming language to calculate both area under curve (AUC) and the top 10 key indicators generated by the model.

### Metagenomic sequencing and analysis

To investigate the bacterial species spectrum associated with NAFLD clinical diagnosis, we conducted metagenomic sequencing on 79 fecal samples obtained from children with NAFLD. The stool specimens were submitted to MyGenostics Co. Ltd. (Beijing, China) for analysis. During metagenomic sequencing, 20 µg of microbial metagenomic DNA was extracted and purified. Also, a sequencing library was prepared using a NEXTflex Rapid DNA Sequencing Kit (NOVA-5144-02, Bioo Scientific, TX, USA) following the manufacturer’s protocols. After library preparation, Qubit 2.0 was used to preliminarily quantify the library, which was then diluted to 2 ng/µL. Agilent 2100 was used to detect the insert size of the library, and the quantitative polymerase chain reaction method was used to accurately measure the effective concentration of the library (>3 mmol/L) to ensure the quality of the library. The constructed library was sequenced on an Illumina NovaSeq 6000 platform. MOCAT preprocessing was employed to ensure the quality of data, which involved filtering out sequences with low quality, insufficient length, or containing errors. The resulting clean data from each sample were then used for metagenomic assembly. Subsequently, all the samples were assembled, with the objective of generating reads longer than 300 bp for further analysis. The genes were combined and clustered using CD-Hit software, ultimately resulting in 2,053,172 nonredundant genes.

The gene catalog was used to compare the sequence with the MicroNR library to obtain species annotation information of unigenes employing bowtie. Subsequently, the gene abundance table was merged to generate a species abundance table with different classification levels. The overall distinctions in microbial composition were evaluated by PCoA analysis of the Bray-Curtis distance. LEfSe was performed, and a significance level of *P* < 0.05 was used to detect significant variations in taxa between patients with NAFL and NASH. The sequences obtained from metagenomic sequencing and the Kyoto Encyclopedia of Genes and Genomes (KEGG) database were compared using BLAST (version 2.2.28+) to identify the metabolic functions of gut microbiota within the samples. The predicted KEGG Orthology-Based Annotation System 2.0 was used for this purpose. The relationship between the structure of intestinal microbiota and NAFLD-related indexes was analyzed using Spearman’s correlation.

### Statistical analysis

The present study used SPSS 22.0 software and an R environment during statistical analysis. Before conducting the statistical analyses, the normality was evaluated for all numerical variables per previous recommendations (16). Generalized linear models were employed to compare continuous data across groups. The chi-square analyses were conducted to compare frequencies among groups, and the independent-sample *t*-tests were used to compare age and BMI across groups. The Kruskal-Wallis test was used to investigate the differences in taxonomic composition and function annotations between groups. Alpha diversity was calculated using the Shannon index. In contrast, differential diversity analyses were conducted using either the Wilcoxon rank-sum test or the Kruskal-Wallis test, depending on the number of groups. Kruskal-Wallis *H*-tests and Welch’s *t*-tests were used to calculate *P* values, and false discovery rates were employed to control for multiple hypothesis testing. Mann-Whitney *U* tests were used to compare the abundance of each phylum and genus among groups. For phyla and genera in which Student’s *t*-tests were employed, the abundance values were ranked and used in regression models. Additionally, each multiple linear regression model was checked for normality using predicted probability plots, and the absence of multicollinearity was assessed using variance inflation factor values.

## RESULTS

### Ecological diversities and significant differences in bacterial abundance between patients with NAFLD and HCs

This study comprised 114 children between 7 and 18 years old, among which 79 were with NAFLD and 35 were HCs. Height, weight, and BMI were measured, and no significant differences were observed in sex or age between the two groups. However, the BMI of patients with NAFLD was significantly higher than that of HCs. The anthropometric and demographic data are presented in Table 2.

**TABLE 2:** Anthropometric and demographic data in NAFLD and HC groups

	HC (*n* = 35)	NAFLD (*n* = 79)	*P*
Sex (male/female)	24/11	65/14	0.064
Age (year)	11.42 ± 1.08	12.23 ± 2.63	0.110
Height (cm)	151.28 ± 14.87	158.22 ± 14.37	0.234
Weight (kg)	40.14 ± 12.11	72.38 ± 20.01	0.090
BMI (kg/m^2^)	17.17 ± 2.34	28.37 ± 4.36	0.001

**Fig 2:**
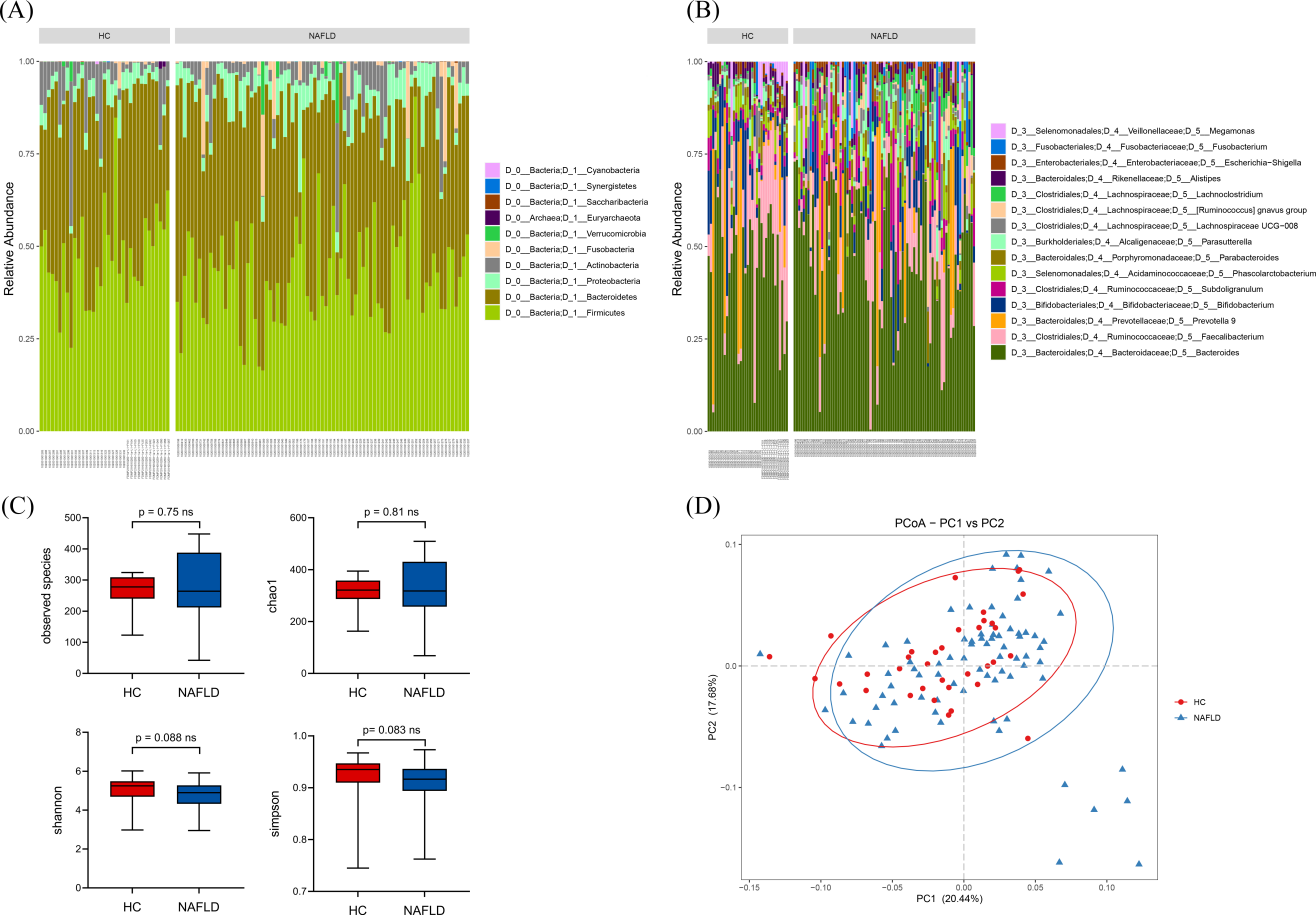
Colony composition and diversity of fecal microbiota in pediatric patients with NAFLD and HCs. (**A**) Top 10 species at the phylum level. (**B**) Top 15 species at the genus level in each sample in pediatric patients with NAFLD and HCs. (**C**) Alpha-diversity group comparison of patients with NAFLD and HCs. (**D**) PCoA of patients with NAFLD and HCs (HC, *n* = 35 and NAFLD, *n* = 79).

The gut microbiomes of 79 patients with NAFLD and 35 HCs were analyzed using 16S rRNA sequencing to explore the differences in intestinal flora diversity and the abundance of various taxa. A total of 16,570,490 sequencing reads were obtained from the 114 samples. The fecal microbiotas observed in the samples were primarily composed of the phyla Firmicutes, Bacteroidetes, and Proteobacteria (Fig. 2A). Only the abundance of Proteobacteria was found to increase in pediatric patients with NAFLD compared with HCs. The dominant genera observed in both groups were *Bacteroides, Faecalibacterium, Prevotella* 9, and *Bifidobacterium* (Fig. 2B). However, the abundance of *Bacteroides* and *Prevotella* 9 both increased in pediatric patients with NAFLD, whereas the abundance of *Faecalibacterium* decreased. Differences in the abundance of several bacteria were observed between NAFLD and HC groups, and these bacteria were planned for further analysis. No significant differences in species richness and evenness were observed between HCs and patients with NAFLD in median alpha diversity (observed species, Chao1, Shannon, and Simpson diversity indices) (Fig. 2C). However, the observed species in patients with NAFLD were more dispersed than those observed in HCs. A PCoA analysis of the gut microbiota samples of the children is presented in Fig. 2D. The statistical models revealed that the composition and abundance of flora species in patients with NAFLD and HCs were similar.

**Fig 3:**
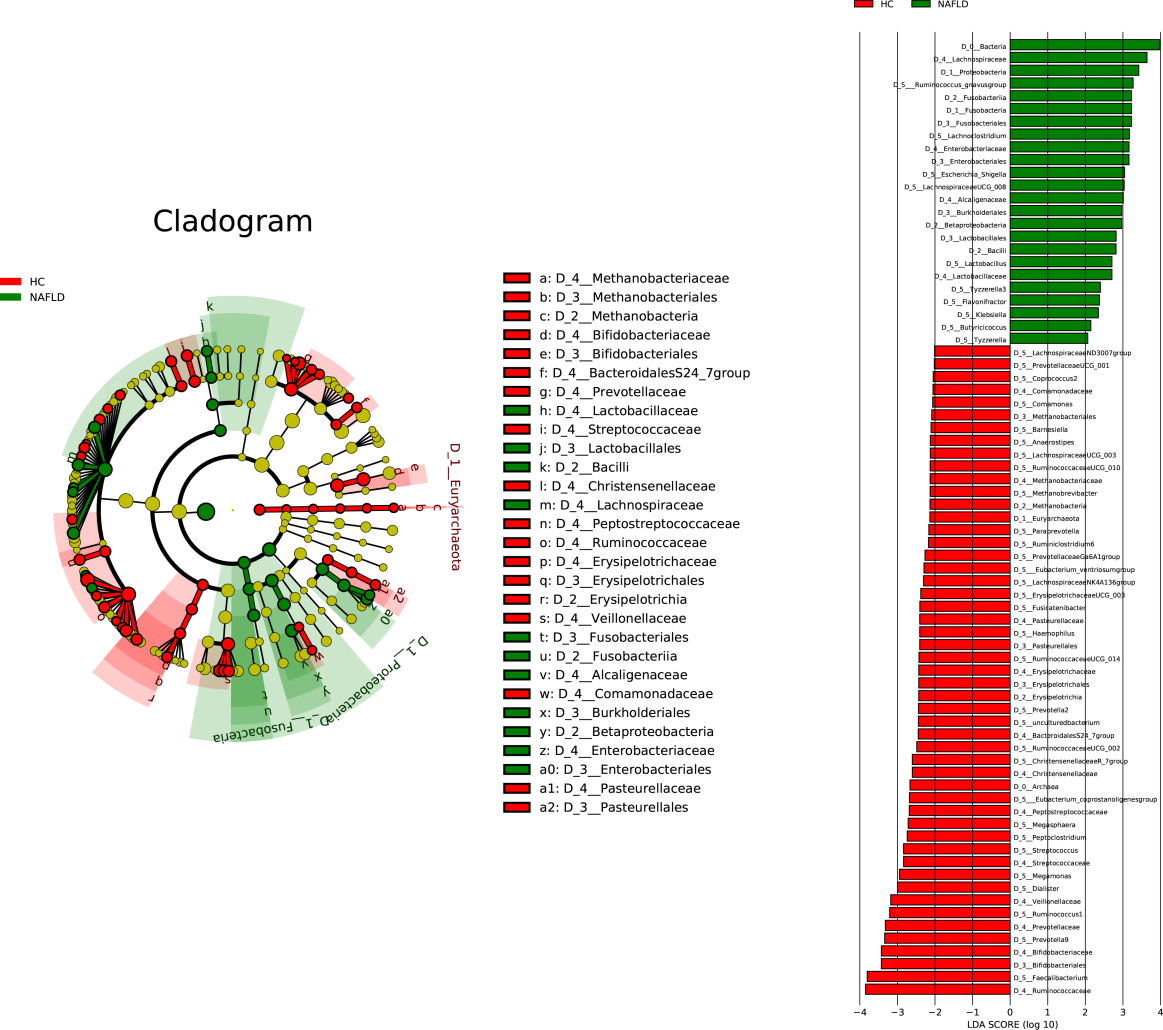
Multiple bacterial species with significantly different abundance were found in the intestinal flora of patients with NAFLD and HCs (HC, *n* = 35 and NAFLD, *n* = 79, *P* < 0.05).

The analysis of bacterial abundance among groups revealed a significant difference in the abundance of several species of bacteria (Fig. 3). Specifically, the abundance of 24 species of bacteria, including Lachnospiraceae, *Proteobacteria, Ruminococcus gnavus* group, *Fusobacteria*, Fusobacteriales, *Lachnoclostridium*, Enterobacteriaceae, Enterobacteriales, and *Escherichia-Shigella,* increased in the gut microbiota of pediatric patients with NAFLD. Conversely, the abundance of 51 species of bacteria, including Ruminococcaceae, *Faecalibacterium*, Bifidobacteriaceae, Bifidobacteriales, *Prevotella* 9, Prevotellaceae, *Ruminococcus* 1, and Veillonellaceae, was significantly lower in patients with NAFLD compared with HCs (Fig. 3). Additionally, *Methanobacteria* belonging to Euryarchaeota was a characteristic genus in HCs. Conversely, *Fusobacteria* was the characteristic genus in patients with NAFLD.

### Ecological diversities and significant differences in bacterial abundance between patients with NASH and those with NAFL

Eight patients with NASH and five with NAFL were identified among children who consented to liver puncture, and the pathological findings were consistent with the diagnosis of NAFLD. Patient BMI, fasting blood glucose, uric acid, TSH, and two liver enzyme indices were measured and compared between the NAFL and NASH groups. Table 3 illustrates the anthropometric and clinical features of the patients. Patients with NASH had higher levels of AST and TSH than those with NAFL.

**TABLE 3:** Anthropometric and demographic data in NAFL and NASH groups^*a*^

	NAFL (*n* = 5)	NASH (*n* = 8)^*b*^	*P* value
Sex (male/female)	4/1	8/0	0.007
Age (year)	11.57 ± 2.23	11.6 ± 3.91	0.023
Height (cm)	152.43 ± 16.48	149.55 ± 11.89	0.386
Weight (kg)	69.20 ± 26.73	57.51 ± 14.37	0.464
BMI (kg/m^2^)	28.80 ± 5.90	25.26 ± 2.50	0.153
Fasting blood glucose (mg/dL)	5.53 ± 1.30	4.98 ± 0.37	0.041
Uric acid (mg/dL)	447.01 ± 79.38	455.5 ± 110.4	0.304
AST (U/L)	54.13 ± 27.07	76.74 ± 61.73	0.591
ALT (U/L)	100.3 ± 61.12	94.67 ± 94.86	0.493
TSH (uIU/mL)	2.76 ± 1.42	3.68 ± 2.43	0.430
FT3	6.44 ± 1.24	6.59 ± 0.71	0.305
FT4	11.63 ± 2.13	11.78 ± 1.64	0.279
NAS	3.14 ± 0.38	4.10 ± 1.10	0.051
Fibrosis stage			
0	2/5	0/7^*b*^	
1a	2/5	0/7	
1b	0/5	0/7	
1c	1/5	1/7	
2	0/5	4/7	
3	0/5	2/7	

^*a*^ FT3, free triiodothyronine; FT4, free thyroxine.

^*b*^ 7 out of 8 NASH patients carried out examined of liver fibrosis.

**Fig 4:**
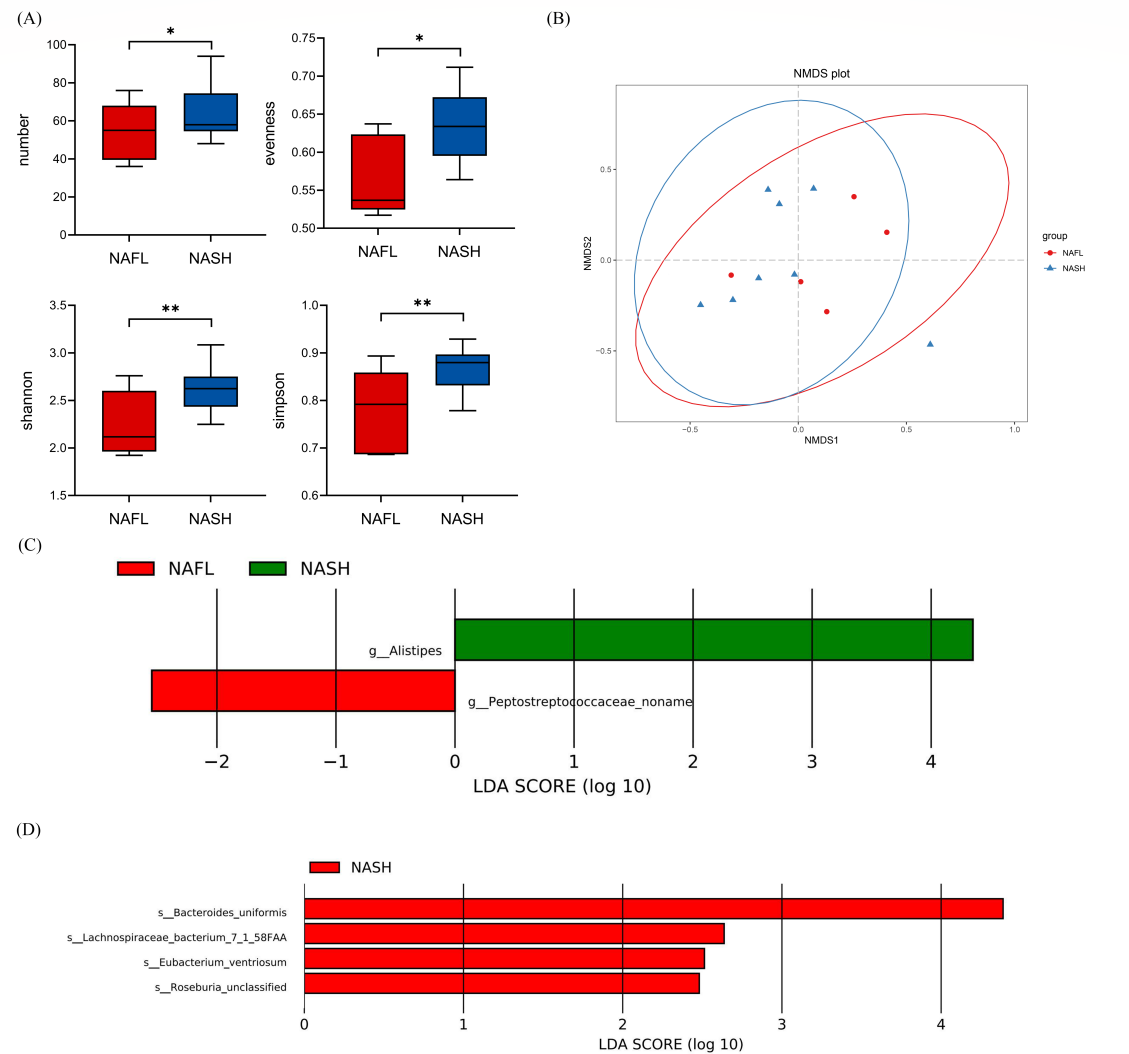
Differential diversity and multiple bacterial species with significantly different abundance were found in the intestinal flora of patients with NAFL and NASH. (**A**) Alpha-diversity group comparison of patients with NAFL and NASH. (**B**) NMDS of patients with NAFL and NASHs. (**C**) Genera with significant differences in abundance between patients with NAFL and those with NASH. (**D**) Species with significant differences in abundance between patients with NAFL and NASH (NAFL, *n* = 5 and NASH, *n* = 8, *P* < 0.05).

The samples from patients with NAFL and NASH underwent metagenomic sequencing to determine the diversity and significant differences in intestinal flora between the two groups. We discovered that the number and evenness of flora species, as well as the Shannon and Simpson diversity indices, decreased in patients with NAFL compared with NASH (Fig. 4A). The non-metric multidimensional scaling (NMDS) plot of the gut microbiota samples revealed that the abundance and composition of flora species in patients with NASH were similar to those in patients with NAFL (Fig. 4B). Further analysis using LEfSe identified several significantly different genera and species between the two groups. Patients with NASH had a higher abundance of *Alistipes,* whereas Peptostreptococcaceae noname had a lower abundance at the genus level (*P* < 0.05, Fig. 4C). The abundance of *Bacteroides uniformis,* Lachnospiraceae bacterium 7_1_58FAA, *Eubacterium ventriosum*, and unclassified *Roseburia* increased significantly in patients with NASH compared with those with NAFL at the species level (*P* < 0.05, Fig. 4D).

### Correlation of bacterial abundance with NAFLD-related indexes

**Fig 5:**
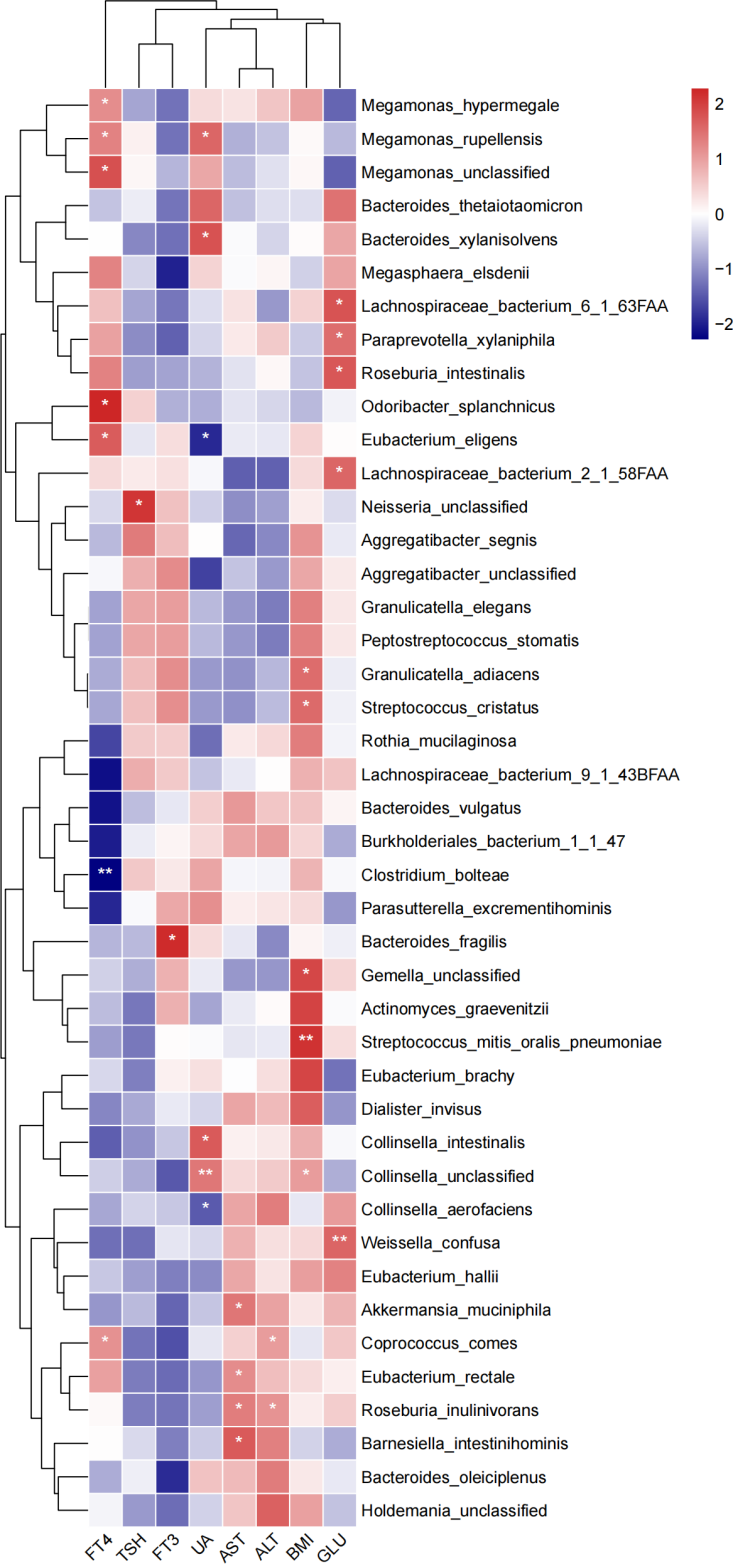
Abundance of species significantly correlated with NAFLD-related indicators at the species level (*n* = 79, *P < 0.05 and **P < 0.01).

We conducted metagenomic sequencing on all 79 NAFLD samples to analyze the correlation between NAFLD-related indices and microbiota in children. The abundance of certain species positively correlated with NAFLD-related indices. The abundance of *Streptococcus mitis, Streptococcus oralis, Streptococcus pneumoniae*, *Gemella* unclassified, *Collinsella* unclassified, *Granulicatella adiacens*, and *Streptococcus cristatus* positively correlated with BMI (Fig. 5). GLU positively correlated with the abundance of *Weissella confusa,* Lachnospiraceae bacterium 2_1_58FAA, *Roseburia intestinalis,* Lachnospiraceae bacterium 6_1_63FAA, and *Paraprevotella xylaniphila* (Fig. 5). ALT positively correlated with the abundance of *Eubacterium ruminantium* group, Ruminococcaceae NK4A214 group, Erysipelotrichaceae UCB-003, *Ruminococcus inulinivorans*, and *Coprococcus comes*. AST positively correlated with the abundance of *Barnesiella intestinihominis*, *Eubacterium redale*, *R. inulinivorans*, and *Akkermansia muciniphila* (Fig. 5). Only the abundance of unclassified *Neisseria* positively correlated with TSH (Fig. 5). Uric acid (UA) negatively correlated with the abundance of *Ruminiclostridium* 9, *Collinsella aerofaciens*, and *Eubacterium eligens*, whereas *Collinsella intestinalis*, *Collinsella* unclassified, *Bacteroidetes xylanisolvens*, and *Megamonas rupellensis* showed a positive correlation (Fig. 5). These findings suggested a correlation between gut microbiota dysbiosis and NAFLD development, with these bacteria being considered NAFLD-related taxa.

### Correlation of bacterial abundance with NAFLD-related metabolic pathways

**Fig 6:**
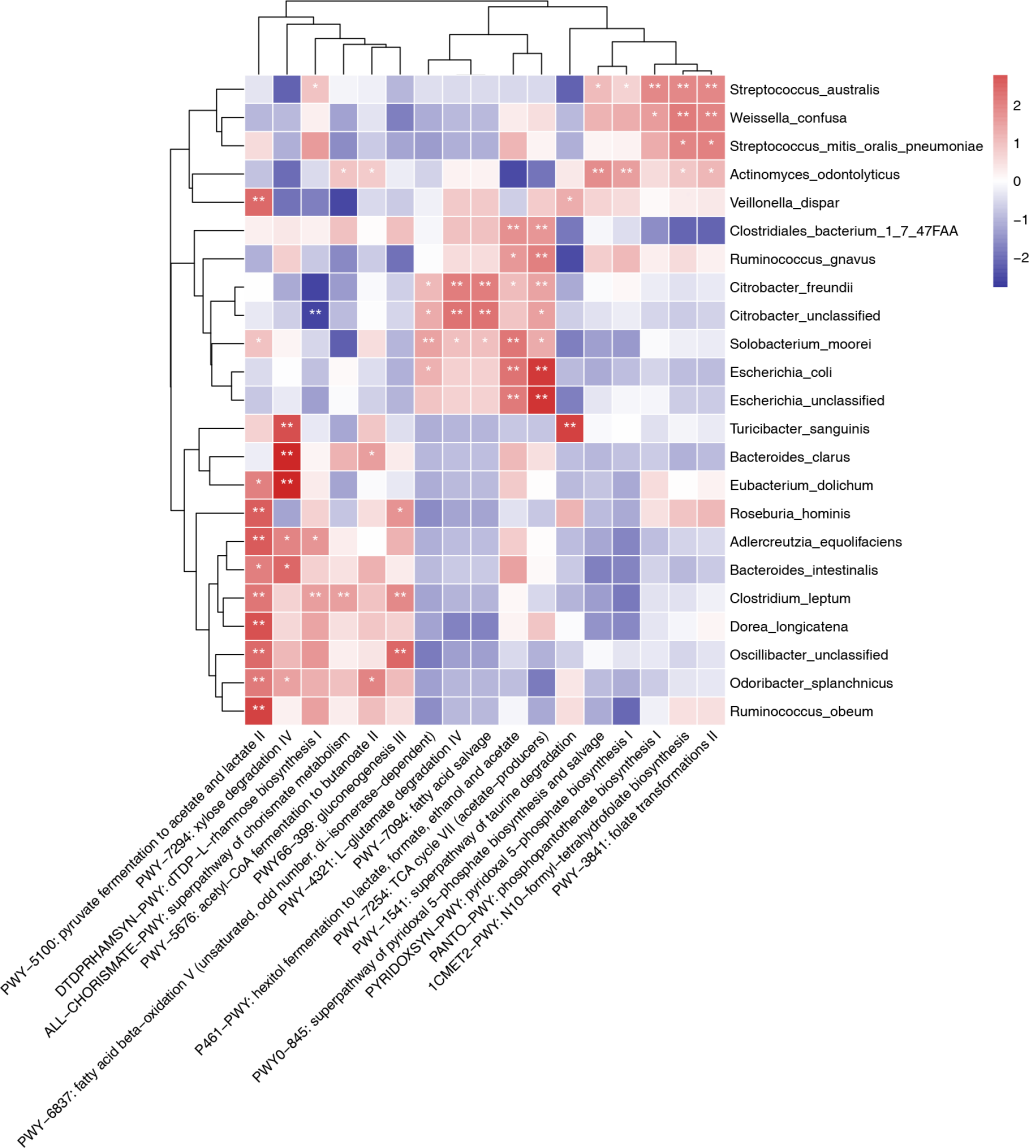
Abundance of species significantly correlated with microbial metabolic pathways potentially related to NAFLD at the species level (*n* = 79, *P < 0.05 and **P < 0.01).

We conducted a further examination of disruptions in gut microflora associated with specific metabolic pathways in NAFLD. These pathways included glucose metabolism, fatty acid metabolism, short-chain fatty acid (SCFA) metabolism, and folate pathways. Utilizing metagenomic sequencing data from 79 NAFLD patients, we assessed the correlation between bacterial taxa and these metabolic pathways. As shown in Fig. 6, the abundance of *Turicibacter sanguinis, Eubacterium dolichum*, and *Bacteroides clarus* was positively correlated with xylose degradation IV (PWY-7294). The abundance of *Weissella confusa, Actinomyces odontolyticus, S. mitis, S. oralis, S. pneumoniae*, and *Streptococcus australis* was positively correlated with N10-formyl-tetrahydrofolate biosynthesis and folate transformations II (PWY-3841), whereas negatively correlated with the abundance of Clostridiales noname. The abundance of *Citrobacter freundii* and *Citrobacter* unclassified was positively correlated with fatty acid beta-oxidation V (PWY-6837), fatty acid salvage (PWY-7094), and L-glutamate degradation IV (PWY-4321). The abundance of *Neisseria subflava, Neisseria flavescens, Fusobacterium periodonticum, Lautropia mirabilis,* and *T. sanguinis* showed a positive correlation with the superpathway of taurine degradation (PWY-1541).

The abundance of different bacterial taxa was strongly associated with the metabolism of SCFAs (Fig. 6). Bacteria such as Clostridiales bacterium 1_7_47FAA, *Escherichia coli*, unclassified *Escherichia*, *Veillonella parvula*, *R. gnavus*, and *C. freundii* were found to be strongly linked to the tricarboxylic acid (TCA) cycle VII, which was involved in the production of acetate. Bacteria such as unclassified *Oscillibacter, Dorea longicatena, Adlercreutzia equolifaciens, Holdemania filiformis, Atopobium, Clostridium leptum, Roseburia hominis, R. obeum, E. dolichum, Odoribacter splanchnicus, Bacteroides intestinalis*, and *Veillonella dispar* were strongly linked to pyruvate fermentation to acetate and lactate II (PWY-5100). The abundance of *Enterobacter cloacae* and *B. clarus* was found to be strongly associated with acetyl-CoA fermentation to butyrate. Similarly, the abundance of *Solobacterium moorei*, unclassified *Granulicatella*, and Clostridiales bacterium 1_7_47FAA was strongly linked to the hexitol fermentation to lactate, formate, ethanol, and acetate (P461-PWY). These findings indicate a close relationship between the composition of fecal bacteria and specific metabolic pathways within the gut microbiota. Perturbations in bacterial composition could potentially impact the development of NAFLD through microbiota metabolism.

### Prediction model of the identified bacterial taxa for the clinical discrimination of patients with NAFLD and HCs

**Fig 7:**
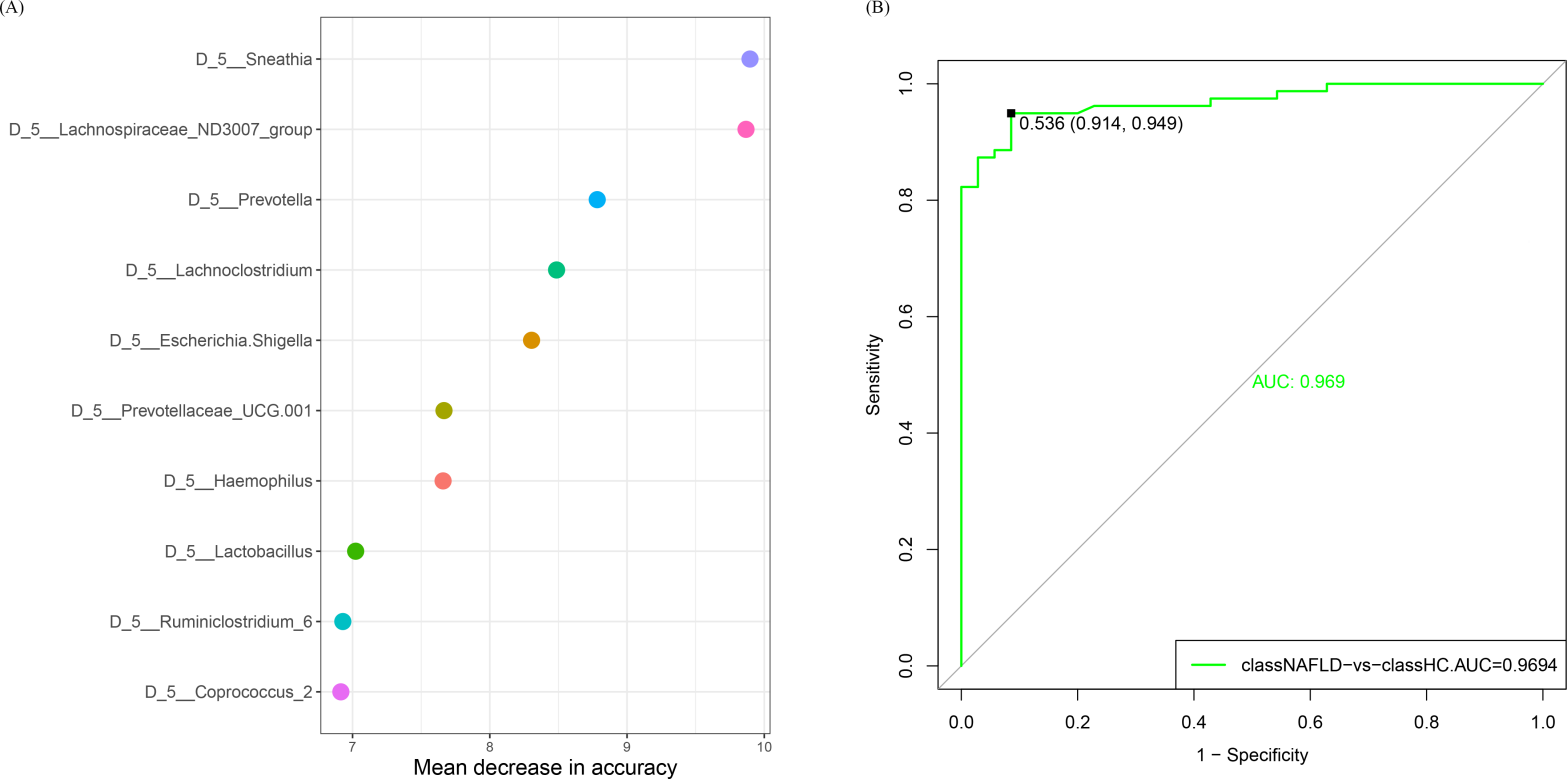
A prediction model of the identified bacterial taxa for the clinical discrimination of patients with NAFLD and HCs was built using the stochastic forest machine algorithm. (**A**) Prediction model constructed using the stochastic forest machine algorithm and (**B**) ROC curves for clinical discrimination.

We developed a stochastic forest machine algorithm to construct a prediction model that could discriminate between patients with NAFLD and HCs based on the identified bacterial taxa. We performed 16S rRNA sequencing analysis of the gut microbiomes of patients with NAFLD and HCs. Ten bacterial taxa, including *Sneathia*, Lachnospiraceae ND3007 group, *Prevotella, Lachnoclostridium, Escherichia-Shigella,* Prevotellaceae UCG-001, *Haemophilus, Lactobacillus, Ruminiclostridium* 6, and *Coprococcus* 2, were selected to predict the risk of NAFLD in children (Fig. 7A). The prediction accuracy of the model was assessed using receiver operating characteristic (ROC) curves, which are presented in Fig. 7B. The model achieved an AUC value of 0.969, with a sensitivity of 0.914 and a specificity of 0.949.

### Relationship of the NAFLD-related changes in bacterial abundance with the BMI under the regulation of nonmedical treatment

**Fig 8:**
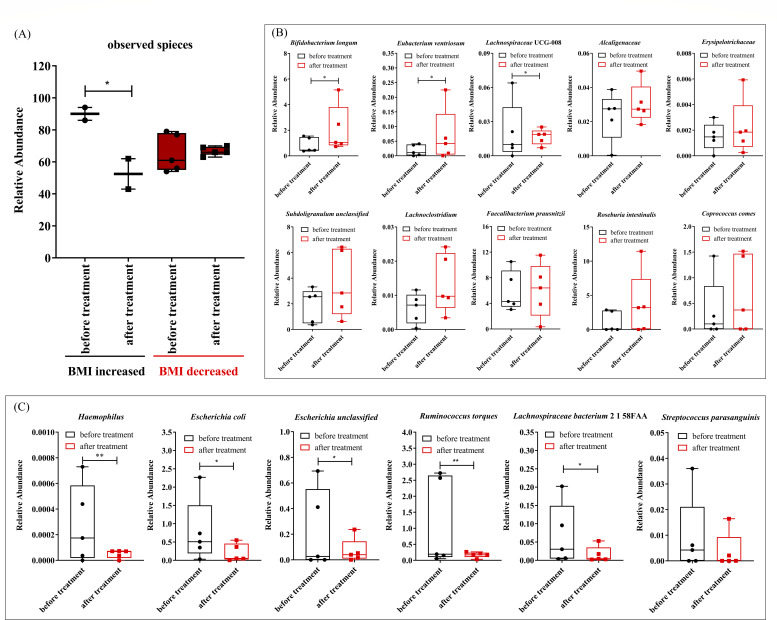
Change in gut microbiota diversity and abundance of NAFLD-related bacteria was closely related to BMI decrease in pediatric patients with NAFLD after nonpharmacological treatment. (**A**) Observed species of bacteria in the flora of pediatric patients with NAFLD. (**B**) Enhanced bacterial abundance and (**C**) reduced bacterial abundance after nonpharmacological treatment.

In this study, we examined the alterations in the diversity of the gut microbiota and abundance of NAFLD-related bacteria before and after nonpharmacological treatment to establish the correlation between gut microbiota and BMI reduction. Fifteen children diagnosed with NAFLD agreed to participate in the study. Five children recorded a decrease in BMI greater than 5% after the nonpharmacological intervention, and their treatment was labeled effective. Conversely, two children had a BMI increase greater than 5%, indicating ineffective treatment. The remaining eight children showed a decrease in BMI of less than 5%, which was deemed irrelevant in this study. Subsequently, we conducted metagenomic sequencing and the resultant analysis of microbial taxa on stool samples of children whose BMI increased or decreased by more than 5%. Figure 8A displays the study results, revealing an increase in the abundance of gut flora species in children with reduced BMI after nonpharmacological treatment. In contrast, those with increased BMI experienced a decrease. The abundance of *Bifidobacterium longum* and *E. ventriosum* increased significantly with nonpharmacological treatment (Fig. 8B). Furthermore, the abundance of Alcaligenaceae, Erysipelotrichaceae, *Faecalibacterium prausnitzii, Subdoligranulum* unclassified, *Lachnoclostridium, R. intestinalis*, Lachnospiraceae UCG-008, and *C. comes* all demonstrated significant increases following nonpharmacological treatment (Fig. 8B). Conversely, the study noted a decrease in the abundance of *Haemophilus, E. coli, Escherichia* unclassified, *Ruminococcus torques*, and Lachnospiraceae bacterium 2_1_58FA in patients with NAFLD with reduced BMI after treatment (Fig. 8C). These results suggested a correlation between the diversity of gut microbiota, the abundance of key species, and the reduction of BMI in NAFLD after nonpharmacological treatment.

## DISCUSSION

The present observational study on children’s health corroborated the hypothesis of a connection between gut microbiota and NAFLD. The stool samples from children diagnosed with NAFLD, NASH, and NAFL, and HCs were analyzed using both 16S rRNA and metagenomic sequencing methods. The analysis aimed to determine the correlation between gut flora, NAFLD-related indicators, and metabolic pathways. The statistical results revealed that the abundance and composition of gut flora in patients with NAFLD were similar to those in HCs. The analysis pointed out that patients diagnosed with NAFLD, NASH, and NAFL, and HCs differed significantly in the bacterial taxa at the genus and species levels. The bacterial abundance had a strong positive correlation with NAFLD-related indexes and metabolic pathways. Notably, the study confirmed the important role of NAFLD-related bacteria in the advancement of the disease after nonpharmacological intervention. Hence, this study unveiled the link between gut microbiota and NAFLD and served as a scientific basis to elucidate the role of diverse bacterial taxa in disease advancement.

Using 16S rRNA analysis, we found that the microbial composition of patients with NAFLD and HCs was significantly different at the phylum, genus, and species levels, with slight differences in diversity between patients with NAFLD and HCs. The abundance of Fusobacteria and Proteobacteria was significantly higher in pediatric patients with NAFLD than in HCs at the phylum level, aligning with the findings of a previous study, which revealed an increase in the abundance of Proteobacteria in overweight patients with NAFLD (17). The upsurge in the abundance of Fusobacteria in pediatric patients with NAFLD was linked to polycellular infections in humans and animals (16). Additional discoveries included the over-representation of potential pathogens in pediatric patients with NAFLD, such as Enterobacteriaceae, *Escherichia-Shigella*, and *Fusobacteria*. Notably, these pathogenic species producing endotoxins could promote liver damage by activating macrophages via the lipopolysaccharide Toll-like receptor (TLR) 4 pathway (18). Our findings aligned with previous findings that established the damaging state of intestinal flora composition in pediatric patients with NAFLD.

We observed lower microbial diversity in patients with NASH and NAFL, as diagnosed using NAS and liver fibrosis stage by liver biopsy. The microbial diversity of patients with NASH was significantly higher than that of patients with NAFL, which might be associated with a higher abundance of multiple bacterial species. Several genera and species, such as *B. uniformis*, Lachnospiraceae bacterium 7_1_58FAA, *E. ventriosum*, *Roseburia*, and *Alistipes*, were significantly more abundant in patients with NASH compared with those with NAFL. In contrast, the abundance of Peptostreptococcaceae noname was lower in patients with NASH. NASH is the active form of NAFLD characterized by hepatic necroinflammation and rapid fibrosis progression (6). Dysbiosis and alterations in intestinal permeability through TLR signaling in hepatocytes induce inflammation progression from simple steatosis to NASH (19). The alterations in the gut microbiota have also been shown to contribute to the development of NAFL/NASH by mediating inflammation, insulin resistance, bile acid secretion, and choline metabolism, among others (20). The abundance of certain species of *Bacteroides*, such as *Alistipes*, which are highly bile tolerant and associated with high-fat diets, negatively correlated with obesity (21). The abundance of Lachnospiraceae bacterium 7_1_58FAA and *E. ventriosum*, both of which produce SCFAs, inversely correlated with body fat percentage (22). A majority of bacteria with a greater abundance in the feces of pediatric patients with NASH were beneficial bacteria. This finding suggested that the microbiome in patients with NASH might present a unique opportunity to interrupt disease progression.

This study demonstrated that the abundance of representative microbial taxa correlated with NAFLD-related indices. This finding suggested that the abundance of bacteria could be associated with NAFLD development. This study found a significant correlation between the abundance of *R. intestinalis* and *R. inulinivorans* and GLU, AST, and ALT levels in NAFLD. A previous study demonstrated a correlation between the abundance of these species and glucose and lipid metabolism in NAFLD (23). Our finding of a positive correlation between TSH levels and the abundance of *Neisseria* unclassified corresponded to a previous study on patients with thyroid nodules, in which the relative abundance of *Neisseria* significantly increased, accompanied by inflammatory disorder (11). Thus, we theorized that these NAFLD-related bacteria might play a crucial role in NAFLD pathogenesis by affecting metabolism. Our study has identified a correlation between the abundance of previously unreported bacterial genera and species and the development of NAFLD in pediatric patients. This discovery provides a scientific foundation for understanding the role of bacteria in pediatric NAFLD and suggests that alterations in the abundance of bacterial genera and species could potentially serve as markers for disease remission.

The functional prediction analysis revealed the involvement of numerous bacterial taxa in pathways related to SCFA production, glucose metabolism, folate metabolism, and fatty acid metabolism. This finding suggested that the gut microbiota might affect the development of NAFL/NASH through energy utilization. The gut microbiota affected the energetic balance of the host by fermenting starch and nonstarter polysaccharides into SCFAs. This process, as previously shown, improved inflammation in NAFL/NASH (24). We identified key species, such as *Oscillibacter* unclassified, *D. longicatena*, *C. leptum*, *R. hominis*, *R. obeum*, *O. splanchnicus*, and *V. dispar*, that were closely related to acetate and butanoate synthesis. Moreover, these species were positively associated with pyruvate fermentation to acetate and lactate II. The abundance of *Clostridiales bacterium* 1_7_47FAA positively correlated with hexitol fermentation to lactate, formate, ethanol, and acetate.

Moreover, the abundance of Clostridiales bacterium 1_7_47FAA, *Ruminococcus gnavus*, and *C. freundii* showed a positive correlation with the TCA cycle VII, which was responsible for acetate production. In contrast, the abundance of *Blautia clarus* positively correlated with the fermentation of acetyl-CoA to butanoate II. *Oscillospira, Dorea, Clostridium, Roseburia, Odoribacter*, and *Ruminococcus* bacteria have been found to be capable of producing SCFAs (12, 25, 26). The reduced abundance of *Roseburia, Ruminococcus*, and *Clostridiales* in pediatric patients with NAFLD indicated their inability to metabolize SCFAs. SCFAs are regarded as a potential target for therapy in managing NAFLD, whereby modulating metabolic processes, preserving the integrity of the gut barrier, and restoring hepatic energy balance can effectively reduce hepatic inflammatory responses (27).

The interaction between SCFA metabolism and intestinal microbiota demonstrated that regulating SCFAs by microbiota had the potential to be a new strategy for treating NAFLD. The abundance of specific gut microbiota strongly correlated with various pathways of glucose metabolism. The abundance of *S. sanguinis*, *Enterococcus faecalis, Clostridium* sp. ATCC BAA 442, and *Blautia clarus*, for instance, positively correlated with the degradation of xylose IV. The coordination between gluconeogenesis and glycolysis was necessary for meeting energy needs under various conditions (28). The results showed that certain bacterial types regulated intestinal glucose metabolism while having the potential to produce SCFAs. The abundance of *W. confusa*, *A. odontolyticus*, *S. mitis*, *S. oralis*, *S. pneumoniae*, and *S. australis* positively correlated with N10-formyl-tetrahydrofolate biosynthesis and transformation of folate II. Reduced endogenous folate levels were observed in obese and diabetic patients, and this deficiency might be related to developing metabolic disorders such as NAFLD (29). Fat mainly accumulates in the liver through the esterification of free fatty acids (FFAs) derived from adipose tissue breakdown caused by insulin resistance in patients with NAFLD (30). In our study, the abundance of *C. freundii* and unclassified *Citrobacter* positively correlated with the fatty acid beta-oxidation pathway V and fatty acid salvage. During infection, host-derived fatty acids are an essential carbon source for pathogenic bacteria (31), and Citrobacter is known as an opportunistic pathogen found in various clinical human specimens (32). We speculated that *Citrobacter*, under nonpathogenic conditions, could utilize FFAs for energy and reduce fatty acid accumulation in pediatric patients with NAFLD. These results illustrated that certain bacterial species in feces had a close relationship with metabolic pathways, and manipulating the gut microbiota composition might significantly impact human health via metabolism. Improving NAFLD progression by manipulating gut microbiota has great potential for future treatment.

The study employed a random forest algorithm to develop a classification model for identifying gut microbiome features in children diagnosed with NAFLD. Identifying such features might aid in the early assessment of NAFLD risk. The classification models based on an AUC value can help design personalized treatment and prevention strategies for NAFLD by facilitating microbiota modulation. The developed classification model had an AUC value of 0.969 for detecting NAFLD risk with a sensitivity and specificity of 0.914 and 0.949, respectively. This study identified gut microbiota features, including Lachnospiraceae ND3007, *Lachnoclostridium* of Lachnospiraceae*, Prevotella* and Prevotellaceae UCG-001 of Bacteroidaceae, *Ruminiclostridium* 6 of Ruminococcaceae, and *Coprococcus* 2 of *Coprococcus* in pediatric patients diagnosed with NAFLD.

This study involved a comparison of the abundance of NAFLD-related bacteria identified in the gut microbiota and the diversity of gut microflora before and after nonpharmacological treatment to assess the impact of altered BMI on an individual’s microbial community. The abundance of gut microbiota species in pediatric patients diagnosed with NAFLD negatively correlated with the BMI after nonpharmacological treatment. This correlation suggested that the gut microflora diversity directly affected the alterations in BMI and might play a crucial role in such cases. Obvious differences in the abundance of NAFLD-related bacteria before and after treatment in children were observed. These differences correlated with the changes in BMI. After a treatment-induced reduction in BMI, the abundance of *B. longum*, *E. ventriosum*, Alcaligenaceae, Erysipelotrichaceae, *F. prausnitzii*, *Subdoligranulum* unclassified, *Lachnoclostridium*, *R. intestinalis*, *C. comes*, and *Bifidobacterium pseudocatenulatum* increased in pediatric patients with NAFLD. Conversely, the abundance of *Haemophilus, E. coli, Escherichia* unclassified, *Ruminococcus torques,* Lachnospiraceae UCG-008, and Lachnospiraceae bacterium 2_1_58FAA decreased. This study confirmed that the abundance of identified gut microbiota in pediatric patients with NAFLD was directly related to BMI. It also found that the bacterial abundance changed with the BMI. Korean adults with NAFLD had a significantly reduced abundance of gut species *Faecalibacterium, Coprococcus,* and Lachnospiraceae (33). In addition, Chinese pediatric patients with NAFLD and those with NASH had a significantly reduced abundance of *F. prausnitzii, Ruminiclostridium,* Bacteroidetes (*Alistipes*)*, Lactobacillus, Oscillibacter,* and *Paraprevotella* species (9, 17). The study consistently found that pediatric patients with NAFLD had a decreased abundance of *F. prausnitzii*, *Lachnoclostridium*, and *Bifidobacterium*. However, the abundance increased significantly after the intervention. Based on previous research on intestinal flora in patients with NAFLD, *F. prausnitzii* and *Lachnoclostridium* were identified as butyrate-producing bacteria (34, 35). Furthermore, the study provided new insights into NAFLD-related bacteria in Chinese pediatric patients with NAFLD and identified several new marker species that were previously unknown, such as *E. ventriosum*, *Haemophilus*, *R. torques*, and *R. intestinalis*. These biomarkers might have a potential prognostic value, might predict overall mortality risk in pediatric patients with NAFLD, and might be useful as biological indicators in future clinical practice.

The present study provided evidence to support our hypothesis that an association existed between gut microbiota and NAFLD. We found that the variations in gut microbiota might potentially play a crucial role in managing NAFLD compared with a single species. Furthermore, we identified several NAFLD-related bacteria, including *Lachnoclostridium*, *Escherichia-Shigella*, and *F. prausnitzii*, whose abundance positively correlated with NAFLD-specific indicators and metabolic pathways. These results revealed new aspects of gut microbiota in Chinese children and adolescents with NAFLD, mainly NASH, and established a microbiota diagnostic profile for NAFLD, opening up therapeutic avenues for probiotics/prebiotics. However, the sample size in this study was relatively small. Hence, further investigations with larger, well-characterized cohorts are needed to better describe the correlation between intestinal flora and NAFL/NASH.

## Supplementary Material

Reviewer comments

## Data Availability

Sequences analyzed in this study are available in GenBank with the accession number PRJNA914785.

## SUPPLEMENTAL MATERIAL

OPEN PEER REVIEW reviewer-comments.pdf An accounting of the reviewer comments and feedback.

ASM does not own the copyrights to Supplemental Material that may be linked to, or accessed through, an article. The authors have granted ASM a non-exclusive, world-wide license to publish the Supplemental Material files. Please contact the corresponding author directly for reuse.

## References

[1] Lee G, You HJ, Bajaj JS, Joo SK, Yu J, Park S, Kang H, Park JH, Kim JH, Lee DH, Lee S, Kim W, Ko G. 2020. Distinct signatures of gut microbiome and metabolites associated with significant fibrosis in non-obese NAFLD. Nat Commun 11:4982. doi:10.1038/s41467-020-18754-5 33020474 PMC7536225

[2] Adams LA, Wang Z, Liddle C, Melton PE, Ariff A, Chandraratna H, Tan J, Ching H, Coulter S, de Boer B, Christophersen CT, O’Sullivan TA, Morrison M, Jeffrey GP. 2020. Bile acids associate with specific gut microbiota, low‐level alcohol consumption and liver fibrosis in patients with non‐alcoholic fatty liver disease. Liver Int 40:1356–1365. doi:10.1111/liv.14453 32243703

[3] Boursier J, Mueller O, Barret M, Machado M, Fizanne L, Araujo-Perez F, Guy CD, Seed PC, Rawls JF, David LA, Hunault G, Oberti F, Calès P, Diehl AM. 2016. The severity of nonalcoholic fatty liver disease is associated with gut dysbiosis and shift in the metabolic function of the gut microbiota. Hepatology 63:764–775. doi:10.1002/hep.28356 26600078 PMC4975935

[4] Mouzaki M, Comelli EM, Arendt BM, Bonengel J, Fung SK, Fischer SE, McGilvray ID, Allard JP. 2013. Intestinal microbiota in patients with nonalcoholic fatty liver disease. Hepatology 58:120–127. doi:10.1002/hep.26319 23401313

[5] Fei N, Bruneau A, Zhang X, Wang R, Wang J, Rabot S, Gérard P, Zhao L. 2020. Endotoxin producers overgrowing in human gut microbiota as the causative agents for nonalcoholic fatty liver disease. mBio 11:e03263-19. doi:10.1128/mBio.03263-19 32019793 PMC7002352

[6] Monga Kravetz A, Testerman T, Galuppo B, Graf J, Pierpont B, Siebel S, Feinn R, Santoro N. 2020. Effect of gut nicrobiota and PNPLA3 rs738409 variant on nonalcoholic fatty liver disease (NAFLD) in obese youth. J Clin Endocrinol Metab 105:e3575–e3585. doi:10.1210/clinem/dgaa382 32561908 PMC7458486

[7] Schwimmer JB, Johnson JS, Angeles JE, Behling C, Belt PH, Borecki I, Bross C, Durelle J, Goyal NP, Hamilton G, Holtz ML, Lavine JE, Mitreva M, Newton KP, Pan A, Simpson PM, Sirlin CB, Sodergren E, Tyagi R, Yates KP, Weinstock GM, Salzman NH. 2019. Microbiome signatures associated with steatohepatitis and moderate to severe fibrosis in children with nonalcoholic fatty liver disease. Gastroenterology 157:1109–1122. doi:10.1053/j.gastro.2019.06.028 31255652 PMC6756995

[8] Del Chierico F, Nobili V, Vernocchi P, Russo A, De Stefanis C, Gnani D, Furlanello C, Zandonà A, Paci P, Capuani G, Dallapiccola B, Miccheli A, Alisi A, Putignani L. 2017. Gut microbiota profiling of pediatric nonalcoholic fatty liver disease and obese patients unveiled by an integrated meta-omics-based approach. Hepatology 65:451–464. doi:10.1002/hep.28572 27028797

[9] Zhu L, Baker SS, Gill C, Liu W, Alkhouri R, Baker RD, Gill SR. 2013. Characterization of gut microbiomes in nonalcoholic steatohepatitis (NASH) patients: a connection between endogenous alcohol and NASH. Hepatology 57:601–609. doi:10.1002/hep.26093 23055155

[10] Zhao Y, Zhou J, Liu J, Wang Z, Chen M, Zhou S. 2019. Metagenome of gut microbiota of children with nonalcoholic fatty liver disease. Front Pediatr 7:518. doi:10.3389/fped.2019.00518 31921729 PMC6933441

[11] Lu LP, Wan YP, Xun PC, Zhou KJ, Chen C, Cheng SY, Zhang MZ, Wu CH, Lin WW, Jiang Y, Feng HX, Wang JL, He K, Cai W. 2017. Serum bile acid level and fatty acid composition in Chinese children with non-alcoholic fatty liver disease. J Dig Dis 18:461–471. doi:10.1111/1751-2980.12494 28585279

[12] Benedict M, Zhang X. 2017. Non-alcoholic fatty liver disease: an expanded review. World J Hepatol 9:715–732. doi:10.4254/wjh.v9.i16.715 28652891 PMC5468341

[13] Kleiner DE, Brunt EM. 2012. Nonalcoholic fatty liver disease: pathologic patterns and biopsy evaluation in clinical research. Semin Liver Dis 32:3–13. doi:10.1055/s-0032-1306421 22418883

[14] Chinese Society of Pediatric Endocrinology and Metabolism, Chinese Pediatric Society, Chinese Medical Association; Chinese Society of Pediatric Gastroenterology, Chinese Pediatric Society, Chinese Medical Association; Chinese Medical Association of Adolescent Medicine, Chinese Pediatric Society, Chinese Medical Association. 2018. Expert consensus on the diagnosis and treatment of Nonalcoholic fatty liver disease in children. Chin J Pract Pediatr 33:487–492. https://link.cnki.net/doi/10.19538/j.ek2018070602

[15] Walters WA, Xu Z, Knight R. 2014. Meta-analyses of human gut microbes associated with obesity and IBD. FEBS Lett 588:4223–4233. doi:10.1016/j.febslet.2014.09.039 25307765 PMC5050012

[16] Zhang X, Hu EA, Wu H, Malik V, Sun Q. 2013. Associations of leg fat accumulation with adiposity-related biological factors and risk of metabolic syndrome. Obesity (Silver Spring) 21:824–830. doi:10.1002/oby.20028 23404933 PMC3661709

[17] Sonnenburg JL, Bäckhed F. 2016. Diet-microbiota interactions as moderators of human metabolism. Nature 535:56–64. doi:10.1038/nature18846 27383980 PMC5991619

[18] Chalasani N, Younossi Z, Lavine JE, Charlton M, Cusi K, Rinella M, Harrison SA, Brunt EM, Sanyal AJ. 2018. The diagnosis and management of nonalcoholic fatty liver disease: practice guidance from the American Association for the study of liver diseases. Hepatology 67:328–357. doi:10.1002/hep.29367 28714183

[19] Ye F, Gao X, Wang Z, Cao S, Liang G, He D, Lv Z, Wang L, Xu P, Zhang Q. 2021. Comparison of gut microbiota in autism spectrum disorders and neurotypical boys in China: a case-control study. Synth Syst Biotechnol 6:120–126. doi:10.1016/j.synbio.2021.03.003 34095558 PMC8163862

[20] Zhang J, Zhang F, Zhao C, Xu Q, Liang C, Yang Y, Wang H, Shang Y, Wang Y, Mu X, Zhu D, Zhang C, Yang J, Yao M, Zhang L. 2019. Dysbiosis of the gut microbiome is associated with thyroid cancer and thyroid nodules and correlated with clinical index of thyroid function. Endocrine 64:564–574. doi:10.1007/s12020-018-1831-x 30584647

[21] Alon-Maimon T, Mandelboim O, Bachrach G. 2022. Fusobacterium nucleatum and cancer. Periodontol 2000 89:166–180. doi:10.1111/prd.12426 35244982 PMC9315032

[22] Carpino G, Del Ben M, Pastori D, Carnevale R, Baratta F, Overi D, Francis H, Cardinale V, Onori P, Safarikia S, Cammisotto V, Alvaro D, Svegliati-Baroni G, Angelico F, Gaudio E, Violi F. 2020. Increased liver localization of lipopolysaccharides in human and experimental NAFLD. Hepatology 72:470–485. doi:10.1002/hep.31056 31808577

[23] Yu J, Zhang H, Chen L, Ruan Y, Chen Y, Liu Q. 2021. Disease-associated gut microbiota reduces the profile of secondary bile acids in pediatric nonalcoholic fatty liver disease. Front Cell Infect Microbiol 11:698852. doi:10.3389/fcimb.2021.698852 34568090 PMC8459332

[24] Stanislawski MA, Lozupone CA, Wagner BD, Eggesbø M, Sontag MK, Nusbacher NM, Martinez M, Dabelea D. 2018. Gut microbiota in adolescents and the association with fatty liver: the EPOCH study. Pediatr Res 84:219–227. doi:10.1038/pr.2018.32 29538359 PMC6185796

[25] Joshi-Barve S, Kirpich I, Cave MC, Marsano LS, McClain CJ. 2015. Alcoholic, nonalcoholic, and toxicant-associated steatohepatitis: mechanistic similarities and differences. Cell Mol Gastroenterol Hepatol 1:356–367. doi:10.1016/j.jcmgh.2015.05.006 28210688 PMC5301292

[26] Guo P, Zhang K, Ma X, He P. 2020. Clostridium species as probiotics: potentials and challenges. J Anim Sci Biotechnol 11:24. doi:10.1186/s40104-019-0402-1 32099648 PMC7031906

[27] Pan X, Kaminga AC, Liu A, Wen SW, Luo M, Luo J. 2021. Gut microbiota, glucose, lipid, and water-electrolyte metabolism in children with nonalcoholic fatty liver disease. Front Cell Infect Microbiol 11:683743. doi:10.3389/fcimb.2021.683743 34778099 PMC8581616

[28] Topping DL, Clifton PM. 2001. Short-chain fatty acids and human colonic function: roles of resistant starch and nonstarch polysaccharides. Physiol Rev 81:1031–1064. doi:10.1152/physrev.2001.81.3.1031 11427691

[29] Takakura T, Francis R, Anani H, Naud S, Raoult D, Bou Khalil JY. 2019. Dorea phocaeensis sp. nov., a new bacterium isolated from the stool of a healthy 29-year-old male. New Microbes New Infect 32:100600. doi:10.1016/j.nmni.2019.100600 31641515 PMC6796640

[30] Yang J, Li Y, Wen Z, Liu W, Meng L, Huang H. 2021. Oscillospira - a candidate for the next-generation probiotics. Gut Microbes 13:1987783. doi:10.1080/19490976.2021.1987783 34693878 PMC8547878

[31] Zhang S, Zhao J, Xie F, He H, Johnston LJ, Dai X, Wu C, Ma X. 2021. Dietary fiber-derived short-chain fatty acids: a potential therapeutic target to alleviate obesity-related nonalcoholic fatty liver disease. Obes Rev 22:e13316. doi:10.1111/obr.13316 34279051

[32] He L, Sabet A, Djedjos S, Miller R, Sun X, Hussain MA, Radovick S, Wondisford FE. 2009. Metformin and insulin suppress hepatic gluconeogenesis through phosphorylation of CREB binding protein. Cell 137:635–646. doi:10.1016/j.cell.2009.03.016 19450513 PMC2775562

[33] Kiess W, Penke M, Sergeyev E, Neef M, Adler M, Gausche R, Körner A. 2015. Childhood obesity at the crossroads. J Pediatr Endocrinol Metab 28:481–484. doi:10.1515/jpem-2015-0168 25928753

[34] Sid V, Siow YL, O K. 2017. Role of folate in nonalcoholic fatty liver disease. Can J Physiol Pharmacol 95:1141–1148. doi:10.1139/cjpp-2016-0681 28460180

[35] Buzzetti E, Pinzani M, Tsochatzis EA. 2016. The multiple-hit pathogenesis of non-alcoholic fatty liver disease (NAFLD). Metabolism 65:1038–1048. doi:10.1016/j.metabol.2015.12.012 26823198

